# Multigene Panel Testing for Hereditary Cancer Risk

**Published:** 2016-05-01

**Authors:** Alyssa A. Grissom, Patricia J. Friend

**Affiliations:** Loyola University Chicago, Marcella Niehoff School of Nursing, Chicago, Illinois

**Multigene Panel Testing for Hereditary Cancer Risk**

A continuing education article for nurse practitioners, clinical nurse specialists, advanced degree nurses, and oncology and hematology nurses.

**Release date:** May 15, 2016

**Expiration date:** May 15, 2017

**Expected time to complete activity:** 0.75 hour

**Meniscus Educational Institute**

3131 Princeton Pike,

Building 1, Suite 205A

Lawrenceville, NJ 08648

Voice: 609-246-5000

Fax: 609-449-7969

E-mail: lrubin@meniscusedu.com

**Journal of the Advanced Practitioner in Oncology**

94 N. Woodhull Road

Huntington, NY 11743

Voice: 631-692-0800

Fax: 631-692-0805

E-mail: claudine@harborsidepress.com

© *2016, Meniscus Educational Institute. All rights reserved.*

## Faculty

**Alyssa A. Grissom, MSN, APN, AGCNS, OCN®**, Loyola University Chicago, Marcella Niehoff School of Nursing, Chicago, Illinois

**Patricia J. Friend, PhD, APN-CNS, AOCNS®**, AGN-BC, Loyola University Chicago, Marcella Niehoff School of Nursing, Chicago, Illinois

## Activity Rationale and Purpose

Historically, an understanding of a patient and/or their family’s cancer risk and predisposition was based in large part on patient pedigree or family history, histology, and age at diagnosis. However, since the completion of the Human Genome Project, advances in technology have moved from pedigree to single-gene testing to multigene testing and next-generation sequencing. Advanced practitioners need to follow developments in genetic testing to interpret results, ensure that their patients/families receive appropriate counseling before and after testing, understand the clinical impact of genetic testing across the trajectory of healthcare, including surveillance, screening, diagnosis, treatment, lifestyle, pharmacogenomics, and new targeted therapies.

Advanced practitioners also need to recognize ethical challenges, limitations, and/or barriers to genetic testing. This article provides an overview of multigene panel testing for hereditary cancer risk and offers two case examples to highlight the clinical considerations, benefits, limitations, and insurance challenges encompassed in this new approach to genetic testing.

## Intended Audience

The activity’s target audience will consist of nurse practitioners, clinical nurse specialists, advanced degree nurses, and oncology and hematology nurses.

## Learning Objectives

After completing this educational activity, participants should be able to:

- Describe the difference between sequential single-gene testing vs. multigene testing
- Discuss the role of multigene testing in hereditary cancer risk
- Explain some of the limitations of multigene testing
- Identify potential barriers to multigene testing regarding insurance coverage

## Continuing Education

**Statement of Credit—Participants who successfully complete this activity (including the submission of the post-test and evaluation form) will receive a statement of credit.**

**Nurses**. This activity for 0.75 contact hour is provided by the Meniscus Educational Institute.

The Meniscus Educational Institute is accredited as a provider of continuing nursing education by the American Nurses Credentialing Center’s Commission on Accreditation.

Provider approved by the California Board of Registered Nursing, Provider No. 13164, for 0.75 contact hour.

## Financial Disclosures

All individuals in positions to control the content of this program (eg, planners, faculty, content reviewers) are expected to disclose all financial relationships with commercial interests that may have a direct bearing on the subject matter of this continuing education activity. Meniscus Educational Institute has identified and resolved all conflicts of interest in accordance with the MEI policies and procedures. Participants have the responsibility to assess the impact (if any) of the disclosed information on the educational value of the activity.

**Faculty**

**Alyssa A. Grissom, MSN, APN, AGCNS, OCN®**, has nothing to disclose.

**Patricia J. Friend, PhD, APN-CNS, AOCNS®, AGN-BC**, has nothing to disclose.

**Lead Nurse Planner**

**Wendy J. Smith, ACNP, AOCN®**, has nothing to disclose.

**Planners**

**Jeannine Coronna** has nothing to disclose.

**Claudine Kiffer** has nothing to disclose.

**Terry Logan, CHCP**, has nothing to disclose.

**Pamela Hallquist Viale, RN, MS, CNS, ANP**, has nothing to disclose.

**Lynn Rubin** has nothing to disclose.

**Content Reviewers**

**Karen Abbas, MS, RN, AOCN®**, has nothing to disclose.

**Wendy J. Smith, ACNP, AOCN®**, has nothing to disclose.

## Disclaimer

This activity has been designed to provide continuing education that is focused on specific objectives. In selecting educational activities, clinicians should pay special attention to the relevance of those objectives and the application to their particular needs. The intent of all Meniscus Educational Institute educational opportunities is to provide learning that will improve patient care. Clinicians are encouraged to reflect on this activity and its applicability to their own patient population.

The opinions expressed in this activity are those of the faculty and reviewers and do not represent an endorsement by Meniscus Educational Institute of any specific therapeutics or approaches to diagnosis or patient management.

## Product Disclosure

This educational activity may contain discussion of published as well as investigational uses of agents that are not approved by the US Food and Drug Administration. For additional information about approved uses, including approved indications, contraindications, and warnings, please refer to the prescribing information for each product.

## How to Earn Credit

To access the learning assessment and evaluation form online, visit www.meniscusce.com.

**Statement of Credit:** Participants who successfully complete this activity (including scoring of a minimum of 70% on the learning assessment and complete and submit the evaluation form with an E-mail address) will be able to download a statement of credit.

## ARTICLE

Cancer is a genetic disease, resulting from germline (inherited) or somatic (acquired) mutations in DNA. Although most cancers arise from acquired DNA damage over an individual’s lifetime, 5% to 10% of cancer diagnoses are caused by an inherited gene mutation (Mauer, Pirzadeh-Miller, Robinson, & Euhus, 2014). Traditionally, genetic testing for hereditary cancer risk follows a sequential single-gene approach through Sanger sequencing methods. Next-generation sequencing (NGS) technology has expanded testing options to include multigene panels.

Multigene panel testing refers to the parallel sequencing of multiple prespecified genes to identify pathologic DNA variation (Hall, Forman, Pilarski, Wiesner, & Giri, 2014). Many cancer susceptibility gene panels are directed toward testing for a specific type of cancer (e.g., Ambry Genetics offers the BreastNext hereditary cancer panel for breast cancer) or a hereditary cancer syndrome (e.g., GeneDx offers the Lynch syndrome/colorectal cancer high-risk panel). Other comprehensive gene panels cast a wider net and include genes implicated in a variety of hereditary cancer syndromes (e.g., Comprehensive Cancer Panel offered by GeneDx).

The identification of an inherited genetic component associated with cancer risk can have a significant impact on risk-reduction and surveillance measures, as well as on treatment decisions if cancer is present. In some cases, multigene panels for inherited cancer risk have proved to be a more time- and cost-efficient approach and are predicted to be increasingly utilized by practitioners.

However, there are a number of issues and challenges to consider when counseling and testing for inherited cancer risk, and they become exceedingly more complex when shifting from single to multigene testing. Technologic advances have outpaced our understanding of the human genome, as genetic variations are being rapidly discovered in which their effect on gene function and clinical significance have yet to be determined.

Genetic counselors play a significant role in recognizing and deciphering the genetic challenges in cancer care. But in clinical practices without genetic specialists, advanced practitioners (APs) are well positioned to facilitate the inclusion of genetics and genomics into patient care. However, currently there is a gap in knowledge and preparation to assume the role in its entirety, especially with the rapid advances in genetic testing technology (Greco, Tinley, & Seibert, 2011). Advanced practitioners must become increasingly proficient in recognizing familial cancer patterns suggestive of hereditary disease, facilitating proper ordering of genetic tests, and interpreting test results, as well as have current knowledge of the ethical, legal, psychosocial, and financial implications of genetic testing for cancer risk. This article provides an overview of multigene panel testing for hereditary cancer risk and offers two case examples to highlight the clinical considerations, benefits, limitations, and insurance challenges encompassed in this new approach to genetic testing.

## OVERVIEW OF MULTIGENE PANELS

Completion of the Human Genome Project has opened the doors for significant clinical advances with a tremendous impact on cancer care, particularly in the ability to detect germline gene mutations that confer an increased cancer risk. Indeed, research has identified mutations in many genes that contribute to more than 50 different hereditary cancer syndromes. Identification of genetic cancer risk has traditionally been done through the application of single-gene testing, but technologic advances have led to the development and application of multigene panel testing, in which a large array of genes can be tested at the same time in a single diagnostic platform to possibly reveal genetic information not available in a single-gene test.

**Figure 1:**
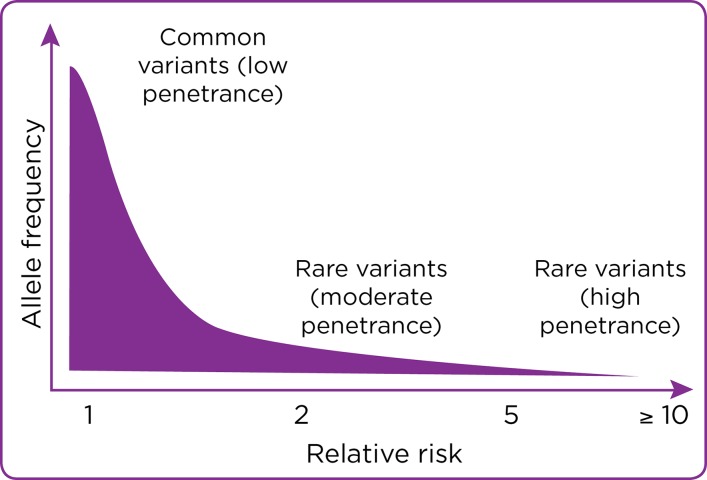
Genetic architecture of cancer risk. Adapted from National Cancer Institute (2015).

Multigene panels consist of a varying number of preselected genes, ranging from 5 to more than 60, and can comprise genes with differing levels of penetrance (see Table 1). Penetrance is associated with disease susceptibility; the higher the penetrance, the greater the risk of disease associated with carrying that gene mutation (see Figure 1). For example, an inherited mutation in a *BRCA1* gene carries a 65% to 70% lifetime risk of breast cancer; thus, a *BRCA1* mutation is classified as a high-penetrance susceptibility gene. Conversely, gene mutations (or variants) with low penetrance are not only more common, but also contribute less to disease risk.

**Table 1:**
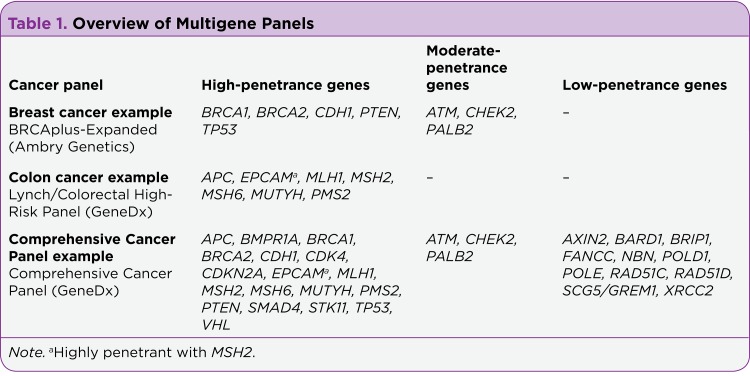
Overview of Multigene Panels

**Figure 2:**
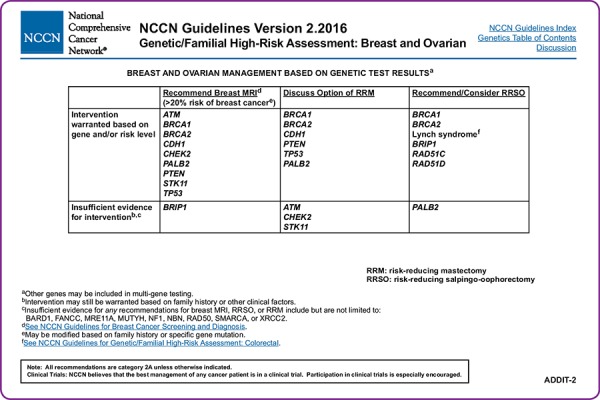
Adapted with permission from the NCCN Clinical Practice Guidelines in Oncology (NCCN Guidelines ®) for Genetic/Familial High-Risk Assessment: Breast and Ovarian Version 2.2016. © 2016 National Comprehensive Cancer Network, Inc. All rights reserved. The NCCN Guidelines® and illustrations herein may not be reproduced in any form for any purpose without the express written permission of the NCCN. To view the most recent and complete version of the NCCN Guidelines, go online to NCCN.org. NATIONAL COMPREHENSIVE CANCER NETWORK®, NCCN®, NCCN GUIDELINES®, and all other NCCN Content are trademarks owned by the National Comprehensive Cancer Network, Inc.

**Figure 3:**
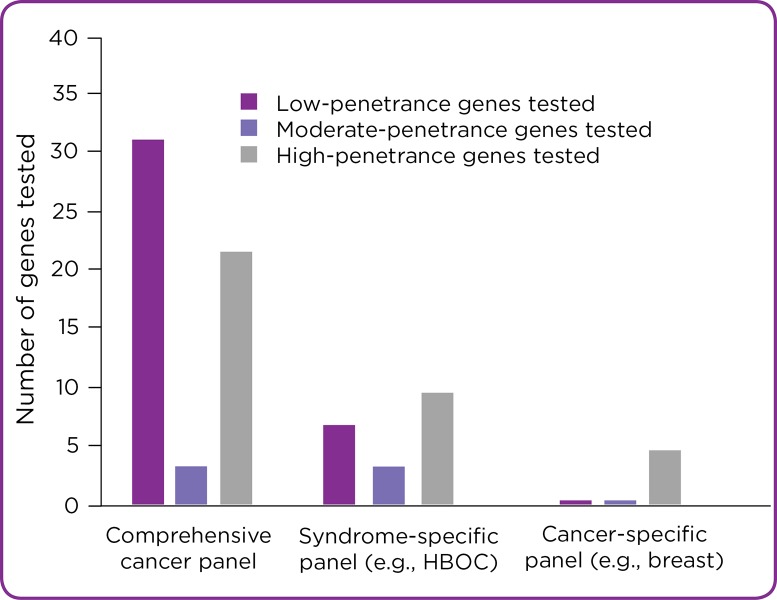
Number of different genes tested according to panel. A comprehensive panel tests more genes but includes more "lowpenetrance" genes, for which clinical significance of identified mutations is unknown. Conversely, a cancer-specific panel tests fewer genes, but the majority of genes have known clinical utility (e.g., high penetrance). HBOC = hereditary breast and ovarian cancer.

The clinical utility of identifying germline high-penetrance gene mutations is strong, as clear recommendations for cancer prevention, surveillance, and management exist within the National Comprehensive Cancer Network (NCCN) Guidelines. Conversely, the clinical utility of identifying germline low-penetrance gene mutations has not been established; thus, clinical practice recommendations are nonexistent for them. Consensus guidelines for screening or management are just beginning to be established for some moderate-penetrance genes (see Figure 2). However, often in these cases, appropriate medical management recommendations with screening or risk-reducing measures are based on patient personal and family histories, in conjunction with other biologic and behavioral risk factors (such as breast density, age, obesity, alcohol intake, and smoking history), more so than genetic test results alone (Fecteau, Vogel, Hanson, & Morrill-Cornelius, 2014; Hiraki, Rinella, Schnabel, Oratz, & Ostrer, 2014). Caution should be taken when performing genetic testing through a multigene panel, which includes a mixed collection of genes with high, moderate, and low penetrance (see Figure 3), as interpretation of results for clinical significance could become a challenge.

The implications of each individual gene tested add to the complexity of multigene panel testing. Gene mutations often increase the risk for more than one type of cancer (i.e., *BRCA2* mutations can lead to an increased risk for breast cancer, ovarian cancer, prostate cancer, pancreatic cancer, male breast cancer, and melanoma). In addition, patients who undergo genetic testing may learn they are at risk for a cancer that has limited or no effectual screening and prevention measures or that invasive surgery (e.g., prophylactic total gastrectomy for CDH1-mutation carriers or bilateral salpingo-oophorectomy for *BRCA1/2*-mutation carriers) is their greatest chance to decrease cancer risk.

Lastly, not all gene mutations are pathogenic, and some genetic tests result in finding variants of unknown significance (VUS), in which practitioners cannot determine whether a patient has an inherited genetic component that increases cancer risk because the pathogenicity of the variant identified is unknown.

## CONSIDERATIONS OF MULTIGENE PANEL TESTING

Advanced practitioners in oncology should be aware of the features of both individual and family histories that are characteristic of an underlying germline mutation (see Table 2). The accurate collection of this information in a complete cancer family history, along with subsequent construction and interpretation of a pedigree, serves as a fundamental tool to perform an assessment of cancer genetic risk. Pedigrees are used to determine inheritance patterns, identify the family member most appropriate for testing, decide upon the appropriate genetic testing options, and create a plan for genetic testing in a family with a positive mutation (Lundy, Forman, Valverde, & Kessler, 2014).

**Table 2:**
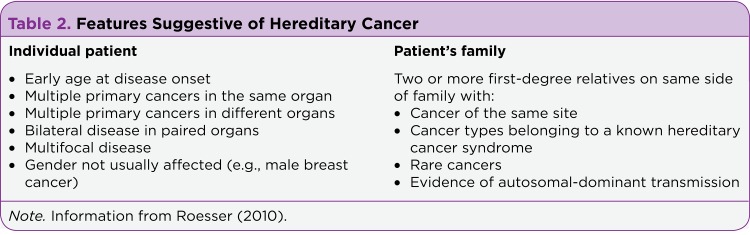
Features Suggestive of Hereditary Cancer

A complete family history should include at least three generations of biologically related family members, with information on ages, cause of death for those deceased, maternal and paternal ethnicities, and cancer history with age of diagnosis at minimum (Wood et al., 2014). For any relative with cancer, the following additional information should also be obtained if at all possible: the primary site of each cancer with pathology reports if available, age at diagnosis for each primary cancer, history of surgery or treatments that may have reduced the risk of cancer (i.e., bilateral salpingo-oophorectomy in a premenopausal woman significantly reduces the risk of ovarian and breast cancers, masking an underlying hereditary predisposition), and carcinogenic exposures (e.g., smoking history).

For relatives not affected with cancer, the following additional information might also aid in pedigree interpretation: history of any surgeries or medical treatments that may have reduced the individual’s risk of cancer, cancer-screening practices, known carcinogenic exposures, and any nonmalignant features associated with the suspected syndrome.

Without having this fundamental information, nongenetic professionals and genetic professionals alike run the risk of misinterpreting hereditary cancer risk.

**Choosing a Genetic Test**

With the clinical application of multigene panel testing, advanced practitioners, on the advice of certified genetic counselors, have the option of choosing one of the following: (1) a single-gene test using Sanger sequencing, (2) a cancer- or syndrome-specific high-penetrant gene panel, (3) a cancer- or syndrome-specific high- and moderate-penetrant gene panel, or (4) a comprehensive cancer gene panel. Multifactorial components can come into play when selecting an appropriate genetic test.

First, one must consider the diagnostic capabilities of sequencing technology used and the suspected type of variation present. Next-generation sequencing may be an appropriate choice for suspected missense, nonsense, or frameshift (insertions and deletions of base pairs) genetic alterations because of its ability to detect single base-pair substitutions, but not for phenotypes commonly caused by trinucleotide repeats or chromosomal abnormalities (Facio, Lee, & O’Daniel, 2014).

Second, multigene panel testing should be considered in certain clinical scenarios (see Table 3). Finally, influences of genetic result turnaround time, patient out-of-pocket cost, insurance coverage, percentage rate for VUS, patient-perceived benefit of testing and interest in expanding testing options, and patient fear of genetic discrimination for insurance and/or employment all need to be considered and discussed with patients during a counseling session to collaboratively come to a decision about testing and which method to employ.

**Table 3:**
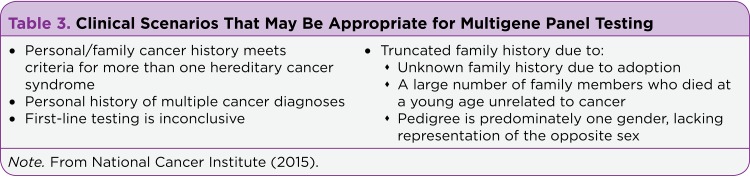
Clinical Scenarios That May Be Appropriate for Multigene Panel Testing

The NCCN Guidelines do not endorse multigene panel testing as a superior option to sequential single-gene testing. However, they do recognize that multigene panel testing may be a more efficient and/or cost-effective option for patients with a personal or family history suggestive of a single inherited cancer syndrome or when more than one gene could explain that inherited cancer syndrome. In addition, the Guidelines state there is a role for multigene testing in individuals who have tested indeterminate for a single syndrome but whose personal or family history remains strongly suggestive of hereditary cancer (NCCN, 2016, p. GENE-1).

Presenting multigene panel testing as an option is often driven by genetic professionals’ clinical judgment and weighing the benefits and limitations with the perceived clinical utility in the setting of a patient’s personal and family histories (Selkirk et al., 2014). Providers can also assess a patient’s perception about genetic risk, motivators for genetic testing, tolerance of probabilistic information or ambiguity in test results, and understanding how results may impact family members to determine whether panel testing would be appropriate (Cameron & Muller, 2009).

## BENEFITS AND LIMITATIONS OF MULTIGENE PANEL TESTING

**Case Study 1: Colon Cancer**

**Figure 4:**
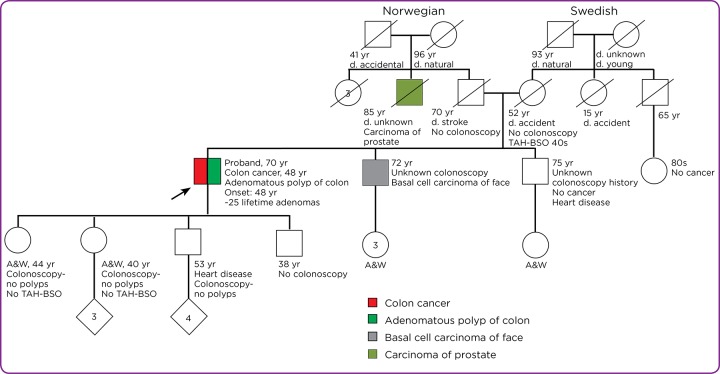
Identification of a PMS2 gene mutation in the proband. TAH-BSO = total abdominal hysterectomy bilateral salpingo-oophorectomy; A&W = alive and well.

The proband is a 70-year-old male with a medical history of colon cancer at the age of 48 and numerous (~25) lifetime adenomatous polyps identified through colonoscopy screening (Figure 4). Pedigree evaluation revealed the following pertinent family history: a paternal uncle with prostate cancer (unknown age of diagnosis) and no other family members with a known cancer or polyp history. At the time of referral for counseling, tumor samples were unavailable, and it was unknown whether microsatellite instability (MSI) and/or immunohistochemistry (IHC) tumor testing was performed at the time of diagnosis.

His personal polyp history and age at cancer diagnosis are the only details suggestive of hereditary disease, with Lynch syndrome (gene mutations in the mismatch repair genes of *MLH1*, *MSH2*, *MSH6*, *PMS2*, or *EPCAM*) or a polyposis syndrome (gene mutations in *APC* or *MUTYH*) suspected. The proband underwent genetic testing with a colon cancer gene panel, which consisted of genes from both syndromes; it resulted in an unexpectedly positive deleterious mutation in *PMS2*.

Multigene panel testing greatly benefited this patient and his family. If a single-gene approach to testing was utilized in this scenario, practitioners would have likely started with the *APC* or *MUTYH* gene based on his adenoma history and then moved on to the mismatch repair genes of Lynch syndrome. It is possible that testing fatigue or cost may have deterred the patient from testing beyond a few genes, and the *PMS2* mutation may have been left undiscovered.

*PMS2* mutations carry a cancer risk of 15% to 20% for colon cancer and 12% to 15% for endometrial cancer compared with that of the general population (ten Broeke et al., 2015; Ambry Genetics, 2016). With identification of a positive mutation in the family, the brother and children of the proband should all consider genetic testing for the *PMS2* mutation, as there are surveillance recommendations for colon and extracolonic cancer risks (refer to NCCN, 2015, p. LS-3 and LS-4). His children are all of appropriate age for colonoscopy screenings and may require them more frequently (every 1–2 years), and his daughters could benefit from increased endometrial cancer surveillance if they are found to carry the *PMS2* mutation.

**Other Benefits**

Costs of gene panel testing continue to decrease due to advances in technology, increasing utilization, and competitive market forces; as a result, it may prove to be a cost-effective option for some patients who could benefit from concurrent analysis of more than one gene. It also reduces the diagnostic odyssey for families meeting testing criteria for more than one hereditary syndrome.

Coverage for genetic testing is left to the discretion of each individual insurance company. Thus, a benefit of cancer multigene panels may be testing for all potential hereditary causes with a one-time insurance review. For example, in the Medicare population, insurance coverage policy restricts genetic testing to just one occurrence; therefore, if patients who had Medicare coverage for *BRCA1* and *BRCA2* analysis and received a negative result for a mutation in either gene, they would have out-of-pocket expenses for any further testing unless they met criteria for a separate hereditary cancer syndrome.

The capabilities of multigene panel testing should be considered with the knowledge of its limitations. Technologic advances have outpaced our understanding of the human genome, as genetic variations are being rapidly discovered in which their effect on gene function are presently unknown; thus, they often produce results that are yet to be clinically actionable.

**Case Study 2: Ovarian Cancer**

**Figure 5:**
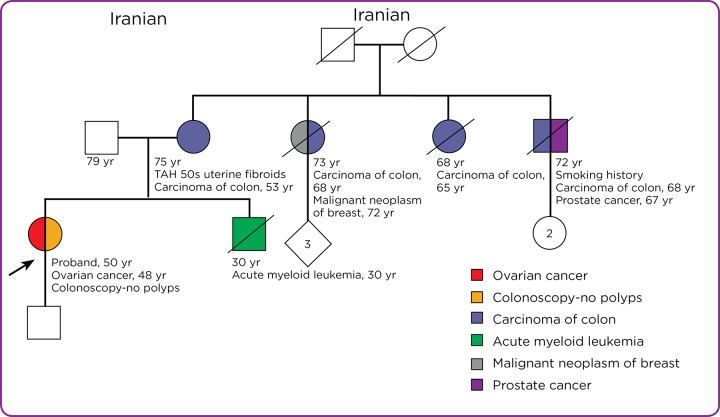
Identification of a CHEK2 variant of unknown significance in the proband. TAH = total abdominal hysterectomy.

The proband is a 50-year-old female who was referred for genetic risk assessment due to a personal history of ovarian cancer, diagnosed at age 48 (Figure 5). Pedigree evaluation revealed the following pertinent family history: four family members with colon cancer, three in their 60s and her mom at age 53; a maternal aunt with both primary colon and breast cancers; a maternal uncle with both primary colon and prostate cancers; and her mom underwent total abdominal hysterectomy with ovaries left intact. The sheer number and spectrum of primary cancers reported in this family are suspicious for a hereditary gene mutation.

The proband decided to undergo genetic testing with a comprehensive cancer panel, and the results identified a VUS in the *CHEK2* gene. For VUS, it is undetermined whether the changes in DNA sequence and structure alter protein and gene function (i.e., the clinical significance of the DNA variation is unknown; Domchek, Bradbury, Garber, Offit, & Robson, 2013).

Unfortunately for this proband and family, with this result, cancer risk and recommendations for screening and risk-reducing measures can only be determined in the context of the family history and any other personal risk factors. Screening recommendations for other cancers (colorectal, breast) should be followed. In the event the *CHEK2* gene mutation had been classified as deleterious, there is a twofold increase in lifetime breast cancer risk, but the level of risk for other associated cancers such as colon, ovarian, kidney, and thyroid is unknown (Rainville & Rana, 2014). The knowledge surrounding genes and their associated cancer risks and the ability to classify variants are constantly evolving. For this clinical case, medical management could one day change as more information becomes available.

**Limitations**

A major limitation of multigene panel testing is that the chance of identifying a VUS is proportional to the number of genes tested (Fecteau et al., 2014). This fact can heighten patient anxiety and distress in addition to the increased chance of discovering cancer risk for which the patient was not expecting and identification of a gene mutation of low clinical utility because penetrance is low or unknown (Kurian et al., 2014).

Next-generation sequencing technology can also have less accuracy than conventional sequencing methods and therefore requires confirmation testing of any pathologic variant before this information can be translated into patient care. Moreover, no matter how advanced sequencing technology becomes, it is not possible to completely rely on standard human reference sequences, because there will always be genomic variation due to common epigenomic changes and intratumour heterogeneity (Javitt & Carner, 2014; Ulahannan, Kovac, Mulholland, Cazier, & Tomlinson, 2013).

Lastly, some commercial health-care insurance plans have labeled genetic cancer susceptibility panels using NGS as investigational and will not provide coverage for it, although the genetic counseling services may be covered. Other insurance plans have identified that all components of the gene panel have to be considered medically necessary following this set of criteria: the genetic disorder is associated with malignancy; the risk of the cancer cannot be identified through biochemical or other testing; a specified mutation or set of mutations has been established in the scientific literature to be reliably associated with the risk of developing malignancy; the results of the genetic test impact the medical management of the individual being tested; the use of the genetic result in directing therapeutic decisions will likely result in an improvement in health and outcomes (Anthem, 2015). Some insurance providers require genetic counseling prior to covering genetic testing services.

**Insurance Challenges and Coverage**

Insurance companies frequently reference the NCCN Guidelines to guide their coverage decisions and as described previously, the NCCN recommends multigene panel testing only in certain clinical scenarios (NCCN, 2016, p. GENE-1). Successful and complete integration of multigene panel testing into clinical practice will require clinical utility evidence, assessing whether the information produced by multigene panel tests improves patient health outcomes (i.e., increases survival) in comparison to conventional sequencing methods. Clinical utility is also the gold standard applied by most payers when evaluating tests for coverage and reimbursement decision-making (Deverka & Dreyfus, 2014).

The current pace of technology development in NGS has left payers with limited ability to determine whether a test meets quality standards (i.e., analytic validity) and clinical utility. Most clinical NGS tests are performed as laboratory-developed tests (LDTs) and do not have direct governmental oversight by the US Food and Drug Administration, in which proponents of this regulation view as a safety concern (Deverka & Dreyfus, 2014). Instead, LDTs are performed in clinical laboratories that have obtained a Certificate of Compliance or a Certificate of Accreditation under the Clinical Laboratory Improvement Act (CLIA) of 1988, which is overseen by the Centers for Medicare & Medicaid Services (Deverka & Dreyfus, 2014; Javitt & Carner, 2014).

To ensure that the quality, accuracy, and reliability of services and the safety of patients are upheld, CLIA aims to measure the analytic validity of any test processed in a certified laboratory. However, there is no current formal CLIA-approved proficiency testing program for NGS, necessitating organizations such as the College of American Pathologists (CAP) and the American College of Medical Genetics and Genomics (ACMG) to develop professional laboratory standards and guidelines (Bennett & Farah, 2014; Deverka & Dreyfus, 2014). The ACMG released standards for NGS in sample preparation, test ordering, test development and validation, staff qualifications, data storage of patient information, and reporting of findings (Rehm et al., 2013).

The certification process for insurance coverage approval of genetic testing is typically submitted by the laboratory. Most of the major laboratories that offer panel testing will notify the patient of out-of-pocket cost greater than $100 to $375, to obtain authorization prior to initiating analysis. In terms of reimbursement, Current Procedural Terminology (CPT) codes related to genetic services fall into either laboratory or professional services. Currently, there are no molecular diagnostic codes that describe NGS sequencing, so gene panels are being billed using nonspecific CPT codes. Given the concerns with lack of transparency in genetic test ordering, the American Medical Association aims to develop NGS-specific CPT codes (Deverka & Dreyfus, 2014).

## IMPLICATIONS FOR ONCOLOGY APS

Advanced practitioners in cancer care utilize many skills of a genetic counselor in their ability to gather a detailed family history, evaluate hereditary cancer risk, and provide psychosocial support. But additionally, they hold responsibility for managing patients’ care from the time of diagnosis, through treatment, and into survivorship or end of life. It is essential for APs to understand how inherited gene mutations can impact medical management decisions for treatment, risk-reduction methods, and surveillance measures, so specific interventions can be incorporated into the plan of care, providing optimal patient outcomes. In current practice, many APs are not credentialed as genetic professionals, often making a referral for genetic risk assessment, counseling, and testing services. However, this practice may not be sustainable with growing public awareness and demand for genetic testing, expanding clinical applications, and declining genetic testing costs.

Advanced practitioners will play increasing roles in the facilitation of genetic services and should engage in a more active role in risk assessment and evaluation of family histories. They can provide genetic education, counseling, and testing within their licensure, scope of practice, expertise, and clinical setting (Greco et al., 2011). The ability to incorporate germline genetic information into patient care is an essential part of oncology practice and essential competency for APs (Robson et al., 2015).

Several studies indicate that health-care providers, who do not specialize in genetics, generally lack the essential knowledge to provide genetic counseling and testing services. Although genetic knowledge may be higher in certain specialty areas, such as oncology or pediatrics, even these providers may have deficiencies with respect to genetic information, especially in light of advancement in genetic testing technology (Bensend, Veach, & Niendorf, 2014). The consequence of having health-care providers unprepared for complex issues related to multigene panel testing and genomics in general may be inaccurate cancer risk assessment and erroneous test selection and result interpretation (Mahon, 2012). These types of errors can result in negative patient outcomes, such as inappropriate medical management, unnecessary risk-reducing surgeries, excessive testing, and psychosocial distress.

Educators across every health-care discipline struggle to incorporate genetic/genomic content into an already-crowded curriculum, and practitioners whose education predated the genomic era are challenged to keep current on changes in genetic technology (Greco et al., 2011). The American Nurses Credentialing Center (ANCC) offers credentialing to nurses’ postgraduate education to obtain board certification as an advanced genetics nurse. Obtaining this level of proficiency will be necessary in the future (Chowdhury et al., 2015), as genetic/genomic science becomes the mainstay of modern medicine.

## CONCLUSION

Multigene panel testing for inherited cancer risk assessment is being rapidly integrated as a new approach to genetic testing. With this technology comes the potential to reveal genetic variations that have uncertainty regarding cancer risk and management decisions. Given the potential issues for patients and their families, multigene panel testing for inherited cancer risk is recommended to be preceded by genetic counseling and in consultation with an experienced genetics professional, which now is starting to incorporate APs. With cancer genomics and genetics leading the field of personalized medicine, oncology APs will need to assume clinical and educator roles to translate genomic/genetic health into clinical practice.
